# Does the Microbiota Composition Influence the Efficacy of Colorectal Cancer Immunotherapy?

**DOI:** 10.3389/fonc.2022.852194

**Published:** 2022-04-08

**Authors:** Yan Lin, De-Xia Kong, You-Ni Zhang

**Affiliations:** ^1^ Health Management Center, Department of General Practice, Zhejiang Provincial People’s Hospital (Affiliated People’s Hospital, Hangzhou Medical College), Hangzhou, China; ^2^ Department of Laboratory Medicine, Tiantai People’s Hospital, Taizhou, China

**Keywords:** colorectal cancer, anti-tumor, pro-tumor, immunotherapy, gastrointestinal microbiota

## Abstract

Colorectal cancer (CRC) is the second most common malignancy globally, and many people with CRC suffer the fate of death. Due to the importance of CRC and its negative impact on communities, treatment strategies to control it or increase patient survival are being studied. Traditional therapies, including surgery and chemotherapy, have treated CRC patients. However, with the advancement of science, we are witnessing the emergence of novel therapeutic approaches such as immunotherapy for CRC treatment, which have had relatively satisfactory clinical outcomes. Evidence shows that gastrointestinal (GI) microbiota, including various bacterial species, viruses, and fungi, can affect various biological events, regulate the immune system, and even treat diseases like human malignancies. CRC has recently shown that the gut microorganism pattern can alter both antitumor and pro-tumor responses, as well as cancer immunotherapy. Of course, this is also true of traditional therapies because it has been revealed that gut microbiota can also reduce the side effects of chemotherapy. Therefore, this review summarized the effects of gut microbiota on CRC immunotherapy.

## 1 Introduction

Colorectal cancer (CRC) is a type of human malignancies associated with the gastrointestinal tract (GI) in which the proliferation and invasion of colon epithelial cells and GI become uncontrollable (1–3). Due to the high metastatic properties of CRC cells and the lack of effective treatment, people with CRC usually die (4). Based on available knowledge, depending on genetics and family history, all people are at risk for CRC, but people over 50 are more likely to develop CRC than other age groups (5, 6). Regarding risk factors for predisposition to CRC, studies have reported that mutations in certain genes, heredity and family history of gastrointestinal malignancies, high-fat and fiber-free diets, obesity and diabetes, gastrointestinal adenomatous polyps, inflammatory bowel disease (IBD), smoking, alcoholism, and GI microbiome composition can increase the susceptibility to CRC (7–10). The origin of CRC is usually polyps formed in the large intestine, which are small noncancerous masses, and over time, under the influence of other predisposing factors, some of these masses can become cancerous tissue (11, 12). For the treatment of CRC, various therapeutic approaches such as traditional therapies including surgery, chemotherapy, radiation therapy, and novel and targeted therapies such as immunotherapy are used to treat patients with CRC (13, 14).

Moreover, the GI is home to trillions of highly diverse microorganisms, collectively termed microbiomes, that can play an important role in regulating biological events and human health (15). It has been shown that the microbiome can be associated with nutrient absorption, digestion and metabolism as well as the host immune system components and responses. Based on recent findings, scientists have discovered that microbiome pattern alteration can lead to diseases such as obesity, diabetes, IBD and cancer (15, 16). It has also been documented that changing the colon environment by manipulating the gut microbiota can reduce the side effects of cancer therapy. For instance, various studies have shown that some probiotic strains can improve chemotherapy-induced mucositis, diarrhea, weight loss. These changes can also reduce inflammation, regenerate and improve intestinal epithelial barriers, and prevent intrinsic apoptosis (17). The microbiota pattern can also be effective in immunotherapy (18). In this regard, researchers have reported that distinct species of *Bacteroides* might be involved in the antitumor effects of cytotoxic T-lymphocyte associated antigen 4 (CTLA4) blockade. Furthermore, specific T cells responses against *Bacteroides fragilis* or *Bacteroides thetaiotaomicron* were associated with greater treatment efficacy with anti CTLA4 (19).

Therefore, considering the importance of the role of microbiota and its possible effect on immunotherapy outcomes, this review discussed the pathogenesis of CRC and its immunopathogenesis and the effect of gut microbial patterns on immunotherapy-based CRC treatment.

## 2 Colorectal Cancer

Colorectal cancer is a disease in which the growth and proliferation of epithelial cells in the colon or rectum is out of control (20). In some cases, the growth of abnormal masses termed polyps in the colon or rectum can cause CRR. To prevent CRC, screening tests can lead to early detection of polyps and allow them to be removed before they become cancerous. Screening tests can also detect CRC in early stages, just as treatment approaches work best (21). Studies have shown that there are different types of CRC, the most common of which is adenocarcinoma. Other types of CRC include gastrointestinal stromal tumors, colorectal lymphoma, and carcinoid tumors.

Additionally, hereditary colorectal cancers are categorized as familial adenomatous polyps (FAP) and hereditary nonpolyposis colon cancer (HNPCC) (22). It has been revealed that aging, especially in the fifth decade, increases CRC risk. One of the most important risk factors is the presence of IBDs, such as ulcerative colitis (UC) or Crohn’s disease. It is also important to have CRC or colorectal polyps, HNPCC, and FAP cases in the family (23). Lifestyle-related factors include lack of regular physical activity, a low-fruit and vegetable diet, a low-fiber and high-fat diet, a diet high in processed meats, overweight and obesity, tobacco and alcohol consumption, and could increase the risk of CRC (8, 23).

In addition, in around 15% of sporadic CRCs, defective DNA mismatch repair (MMR) could be occurred. Several retrospective investigations have shown that MMR-deficient CRC patients have a more promising stage-adjusted prognosis than those who have MMR-proficient CRC (24).

Based on the findings of previous studies, colorectal polyps and CRC are not always asymptomatic, especially at the beginning of the disease. Many people with CRC or polyps may not be aware of the disorder, so regular screening tests are essential for the early detection of CRC (25). Clinical signs of CRC usually include changes in bowel habits, blood in the stool, diarrhea, constipation or feeling of complete bowel emptying, chronic abdominal pain or cramping, as well as unexplained weight loss (26). Therefore, it is recommended that adults 45 to 75 years of age be screened for CRC. Screening tests, including periodic stool tests, CT colonography, and flexible sigmoidoscopy, can help diagnose polyps or CRC (27).

It has been revealed that the early-onset CRCs show differential molecular, clinical, and pathological features than later-onset CRCs (28). Early-onset colorectal cancer (CRC) occurs in people below 50 years has been growing worldwide, particularly in high-income countries (29). It has been reported that the exposome and early-life environmental exposures, including western diets, red and processed meat, obesity, antibiotics, stress, synthetic dyes, monosodium glutamate, titanium dioxide, high-fructose corn syrup, birth mode, breastfeeding behaviors, and maternal stress, could affect microbiome health and development (30). Moreover, some of the mentioned exposures may lead to genetic and epigenetic modifications in CRC cells and affect the gut microbiota and host immunity. Additionally, the gene-by-environment interactions throughout life may play a pivotal role in the early-onset CRC etiology. Moreover, hypomethylation of long-interspersed nucleotide elements1 (LINE1) is sporadically detected in CRC (31). A wide range of exposures such as physical inactivity, high BMI, pesticides, ionizing radiation, smoking, benzene have been accompanied by LINE1 hypomethylation in blood cells (32–34). Regarding the relationship between tumor LINE1 hypomethylation and younger age at CRC diagnosis, it is probable that gene-by-environment interactions along with mentioned exposures, particularly in early life, play an etiological role in early-onset CRC (35, 36). Therefore, evaluating early-life exposures, gene-by-environment interactions, and genetic germline polymorphisms could improve the screening effectiveness and early diagnosis of early-onset CRC (28).

These data collectively indicated that improving lifestyle and diet to develop a healthy microbiota may contribute to early-onset CRC prevention and a more favorable treatment response.

## 3 Immune System and Colorectal Cancer

As we know, the TME of solid tumors has complex properties and special conditions, and due to the presence of hypoxia, high acidity, different signals as well as the presence of immune system cells, different mediators, endothelial cells, fibroblasts and other growth spectrum cells provides tumor cell development (37, 38). Immune cells in the TME can have pro and antitumor roles based on their phenotypes (39, 40). The main components of the innate immune system include physical epithelial barriers, circulating plasma proteins, phagocytes, dendritic cells (DCs), natural killer (NK) cells, and lymphatic cells. These innate immune system components are usually present in all tissues (41). However, understanding the function of these cells in the TME remains unknown (42). For instance, researchers have long believed that NK cells are an effective antitumor agent in CRC, but novel investigations have shown that despite the secretion of chemokines and adhesive molecules associated with the recruitment and homing of NK cells in tumor tissue, there is a surprising absence of these cells are in the TME (43–46). Correspondingly, due to the predominance of myeloid cells over lymphocytes and even tumor cells, different myeloid cell origins and unknown differentiation programs for myeloid subtypes add to the complexity of understanding the components of innate immune function in the site of tumor (47–49).

Chronic inflammation subsequent from tumor function or the immune system to control the tumor is one of the hallmarks of human malignancies such as CRC, which promotes and maintains cellular transformation and tumor development in CRC (50). The source of this chronic inflammation can be persistent infections, autoimmunity, and sterile inflammation, in all of which the innate immune system cells are the main performers (51). Although the extent of these cells’ involvement in the initial inflammatory response is not fully understood, the TME’s signals intelligently manage these innate immune cells and their mediators in favor of the cancer development. Inflammatory signals include heat shock proteins (HSPs) and toll like receptors (TLRs), apoptotic cells, damage-associated molecular patterns (DAMPs), cytokines, or free DNA molecules that are responsible for dysregulation of immune system responses (52–55). Following these signals, the secretion of chemokines leads to the recruitment of more immune cells to the TME, which can act as a suppressor or activator of antitumor responses and ultimately lead to tumor development or inhibition (38).

As mentioned, the distribution and abundance of immune cell subpopulations in the TME of solid tumors are very diverse. In this regard, it has been found that there is a close relationship between the number of immune cells and the clinical course of CRC (56–58). For example, the high frequency of effector T cells with antitumor properties is associated with a good prognosis, and conversely, the high frequency of infiltrated myeloid cells in TME is associated with a poor prognosis leading to tumorigenesis (59–61). Evidence suggests that the adaptive immune system components, including T cells and B cells, play an important role in CRC and can learn to detect tumor cells and contribute significantly to the course of the disease (38). The presence of effector T cells in the TME is typically considered a sign of inflammation, while regulatory T cells (Tregs) are considered a sign of immunosuppression (62–64). However, in CRC, the high frequency of Tregs is associated with a better prognosis, which contradicts the negative association of FOXP3^+^ tumor-infiltrated Tregs in other cancers (61). In CRC metastatic liver lesions, the composition of the TME is mainly determined by the chemokine and cytokine pattern, and regularly a small number of FOXP3^+^ Treg and NK cell cells are recruited to the tumor site (65). It has been reported that a small number of CRC patients with unstable microsatellites (MSIs) tumors that contain impaired DNA repair-related proteins, immunity-related mutations are increased, resulting in a remarkable upsurge in the presence of adaptive antitumor immune cells such as lymphocytes, leading to promising prognosis. While the number of infiltrated cells in microsatellite stable CRC tumor tissue (MSS) is less than half, these patients respond to immunotherapy much better than the MSI subgroup (66, 67).

Since the different phenotypes of different B cells, the role of these cells in the CRC TME is highly controversial (68–70). The formation of tertiary lymphoid structures seems to occur with the juxtaposition of B cells and T cells in CRC, indicating an increase in effector immune cells’ penetration and, ultimately, a more favorable prognosis. However, some studies show that mutations in the B-Raf proto-oncogene, serine/threonine kinase (*BRAF*) gene are associated with the formation of tertiary lymphoid structures (71–74).

The tumor escape mechanisms from the immune system are very diverse and intelligent. Tumors can induce the production of cytokines that enhance Tregs and MDSCs and inhibit the function of CD8^+^ cytotoxic T cells. These occurrences can lead to the suppression of CD4^+^ and CD8^+^ effector T cells that can no longer detect or respond to tumor antigens. Tumor cells can also reduce the expression of MHC-I so that T cells can no longer recognize these cells. In addition, tumors can induce the expression of immunosuppressive molecules such as CD274 (PDL1), CTLA4 (CD152), lymphocyte activating 3 (LAG3), and T-cell immunoglobulin domain and mucin domain 3 (TIM3), which lead to exhaustion of effector T cells as well as inhibition of malignant cell apoptosis (75–77). Targeting these same immune checkpoint molecules forms the basis of immunotherapy using the blockers of these inhibitors.

Evidence designates that lifestyle, diet, nutrition, the microbiome, the environment, and other exogenous factors could be responsible for the formation of pathologic states and affect the genome, epigenome, transcriptome, proteome, and metabolome of cancerous and noncancerous cells, as well as immune cells. Nowadays, the importance of studying big data is felt more and more, and this requires the transformation of pathology to epidemiology, biostatistics and bioinformatics data science fields. The Molecular Pathological Epidemiology (MPE) research framework is able to reveal the advantages and strengths of interdisciplinary integration that has been used to study several human malignancies such as CRC, lung, breast, and prostate cancers. The MPE research model offers new insights into the interactions between the environment, the tumor, and the host and could open new research frontiers (78).

In this context, the combination of tumor immunology assessments with the MPE approach can evaluate the impact of endogenous and exogenous factors on tumorigenic processes leading to CRC by assessing antitumor immune responses (79, 80). This integrative immuno-MPE field is able to fill a research gap between epidemiology and tumor immunology, and it signifies a future direction for cancer investigations (79, 81, 82). The immune status analyses in the TME are gradually being unified into large-scale epidemiological cohorts (83–88).

## 4 Immunotherapy for Colorectal Cancer Treatment

Based on studies on the use of immunotherapy-based methods in the treatment of patients with CRC, the success of these methods compared to traditional therapies can be recognized to some extent, although sometimes for different reasons and specific conditions of tumor cells and TME along with the unpredictable behavior of the immune system, immunotherapy also faces major challenges (76). Immunotherapy in MSI-H or dMMR patients has been performed and approved, and currently, immune-checkpoint blockers such as programmed cell death 1 (PDCD1) inhibitors are being studied more than other approaches. About 80% of cases of MSS or MMR-p can be detected in the pathology of advanced stages of metastatic CRC, and it is possible that combination therapies using immune-checkpoint blockers and other tumor-inhibiting factors such as radiotherapy, chemotherapy, and cancer vaccines together can lead to strong antitumor responses from the patient’s immune system (76, 89, 90). However, combining these methods has not led to satisfactory clinical outcomes in several studies (91).

Therefore, studies are not limited to the use of immune-checkpoint blockers and other treatments such as chimeric antigen receptor (CAR) T-cell therapy and cancer vaccines, as well as interventions in the microbiome pattern of patients with CRC evaluated.

CAR-T cells, especially the third generation and later, have successfully treated human blood malignancies such as acute B-cell lymphoblastic leukemia by targeting specific antigens (92, 93). However, in solid tumors, due to various problems such as lack of penetration and ineffective trafficking, the tumor inhibitory TME has not yet achieved satisfactory consequences (94). In metastatic CRC mouse models, targeting the guanylate cyclase 2C (GUCY2C) antigen, expressed by tumor cells, could eliminate these cells and inhibit metastasis (95). Clinical trial studies are also underway on CAR-T cells in treating patients with CRC. In this context, targeting the epidermal growth factor receptor (EGFR) antigen and the design of CAR-T cells against this receptor are underway (NCT03152435). Despite the challenges, with the advancement of science and the identification of a wider range of tumor-specific antigens, a moral prospect for this treatment for CRC can be envisioned.

Vaccine therapy is another type of immunotherapy that can also treat cancer. Based on current theories and backgrounds of vaccines and their effect on the innate and adaptive immune system, cancer vaccines may lead to more effective antitumor responses by inducing tumor-related antigens to be targeted by the immune system (76, 96, 97). Various studies have been performed on anti-metastatic CRC vaccines, including peptide vaccines, DC vaccines, and autologous vaccines (98–100). However, the available evidence suggests that cancer vaccines are generally not as effective as other immunotherapy-based methods and traditional therapies such as chemotherapy and surgery in increasing patients’ survival for a variety of reasons, including the genome-wide tumor mutations and the development of neoantigens, as well as high tumor heterogeneity (101, 102).

## 5 Role of Gut Microbiome in Colorectal Cancer

**Figure 1:**
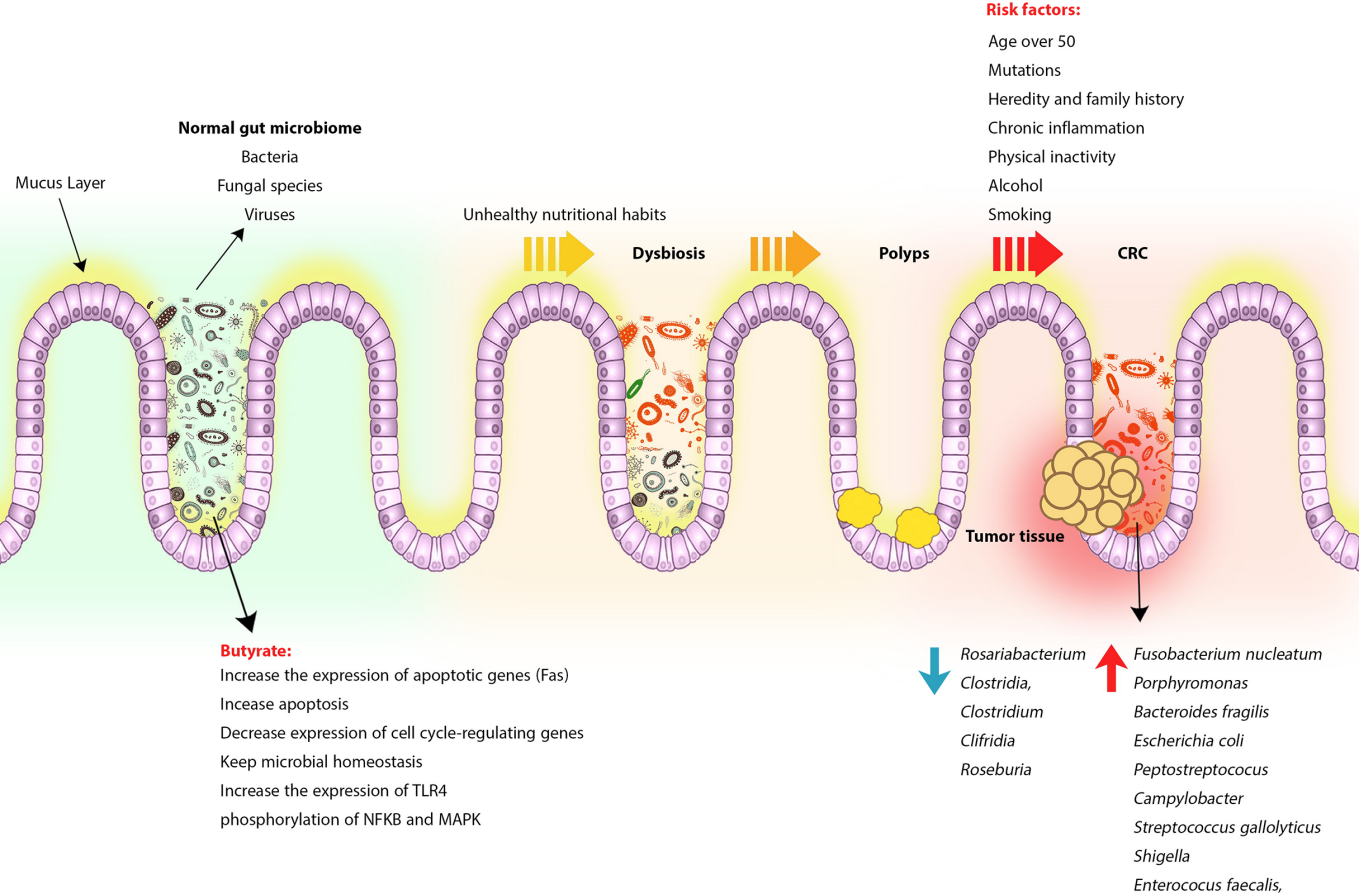
CRC formation process following gut microbiome change and dysbiosis. Normally, the gut microbiome, made up of bacteria, viruses, and fungi, contributes to intestinal homeostasis and immune regulation by producing beneficial metabolites such as butyrate and SCFA, but following a change in lifestyle and poor eating habits, consuming high-fat and low-fiber foods, red meat and processed foods alter the intestinal microbiome pattern and replace harmful and tumorigenic species. Dysbiosis can lead to chronic inflammation, polyps, and eventually CRC. However, other risk factors can also play a role in increasing the risk of developing CRC.

As mentioned earlier, genetic and environmental factors play an important role in increasing the incidence of CRC (103). In developing countries, changing lifestyles and unhealthy nutritional habits such as low-fiber or high-fat diets, consuming processed meats and red meat, along with physical inactivity, alcohol and smoking, can alter the gut microbiota pattern and influence several physiological processes, immune system responses, and even cancer treatment (104–107). Contrary to popular belief, the gut microbiome is not limited to bacteria, but fungal species and viruses are also components of the gut microbiome. For example, the DNA load of the virus in tumors is much higher than in noncancerous tissue. Studies of viral infections such as human poliovirus, human herpes, and human papillomavirus have also shown that these viruses can be involved in the pathogenesis and risk of CRC (108, 109) (**Figure 1**).

### 5.1 Effect of Diet on Gut Microbiome

Evidence suggests that diet can reduce or increase the risk of various diseases, including CRC (110). A prudent diet may protect against stroke, cardiovascular diseases (CVDs), and other frequent disorders such as gastrointestinal cancers (111). This diet contains vegetables, fruits, fish, legumes, whole grains, nuts, and low-fat dairy foods rather than processed or refined foods, butter, egg, red meats, and high-sweet products. In this regard, it has been reported that foods containing fiber, such as whole grains, are among the most effective factors associated with reducing CRC risk (112–114). However, the epidemiological data have significant heterogeneities due to the prudent dietary patterns and the main components of the prudent diet associated with CRC (113).

On the other hand, according to previous studies, Western diets containing high-fat, high salt, and processed meats have been linked to CRC (114, 115). However, the mechanisms of this correlation are not yet fully understood (110). A theory is that the gut microbiota may play a mediating role because changing dietary habits from a prudent diet to a high-fat, high-salt, low-fiber diet significantly enhances the bacterial and metagenomic profiles of the gut microbiota, such as an increase in the stool *Fusobacterium nucleatum* (*F nucleatum*) levels, resulting in elevation of inflammation-related metabolites serum levels (110, 116, 117). Prospective cohort studies reported that subjects with a long-term prudent diet (high fiber) were accompanied by a lower risk of *F nucleatum* ^+^ CRC but not *F nucleatum* ^-^ CRCs. These findings could also indicate the possible effects of prudent fiber-rich diets by modulating levels of specific bacterial species such as *F nucleatum* in preventing CRC (118).

### 5.2 Effect of Lifestyle and Other Environmental Factors on Gut Microbiome

The intestinal microbiota has been shown to be associated with obesity, diabetes, IBD, cancer, rheumatoid arthritis (RA), and CVDs (119, 120). On the other hand, other factors such as age, host sex, genetics, diet, drugs, smoking, alcohol consumption, and living environment influence the microbiota pattern (121).

#### 5.2.1 Smoking

Evidence has shown that smoking is associated with alterations in gut microbiota pattern, IBD, and especially *Clostridium difficile* infection (122, 123). Before and after 4 to 10 weeks of smoking cessation, smokers’ intestinal microbiota can undergo significant changes (124). On the other hand, animal studies also confirm a relationship between the immune system and chronic cigarette exposure because smoking can lead to changes in the mucosal immune system in the intestine (121, 125). Analysis of the gut bacterial pattern of current smokers showed a decrease in *Firmicutes* and *Proteobacteria* and an increase in *phylum Bacteroidetes* levels compared with never smokers. While there were no differences between bacterial gut patterns of former and never smokers (126). These findings clearly show the effect of smoking on changing the pattern of gut microbiota.

#### 5.2.2 Alcohol Abuse

The intestinal flora plays an important role in the pathogenesis of alcoholic liver damage (127, 128). On the other hand, alcohol abuse is the most common cause of liver disease in Western countries, changing the amount and composition of gut microbiota. Although the precise mechanism(s) of these changes following alcohol abuse is not well elucidated, it appears that following mucosal damage and increasing gut permeability, bacterial products translocation into the portal blood could be responsible for alcohol-induced liver damage by inducing the release of inflammatory mediators, including interleukin 1 beta (IL1B), tumor necrosis factor (TNF), chemokines, leukotrienes, and reactive oxygen species (ROS), increasing inflammatory responses and fibrosis in the liver, and other body organs (129, 130). Studies in this area have reported that gut microbiota manipulation is a potential therapeutic approach to reduce liver damage caused by alcohol abuse (127). However, more studies are required to confirm the therapeutic effects of gut microbiota modulation in liver damage and other disorders.

#### 5.2.3 Exercise

Studies in athletes have shown that exercise can enrich the diversity of intestinal microflora, especially *Faecalibacterium prausnitzii*, preserving a healthier intestinal environment (131). Although diet and exercise are effective in this regard, exercise alone can also lead to increased intestinal microbial diversity. Interestingly, the frequency of butyrate-producing species, such as *Erysipelotrichaceae, Roseburia*, *Lachnospiraceae*, and *Clostridiales*, increased in the gut microbiome following exercise (132). It has also been shown that exercise, as therapeutic support, can be helpful for dysbiosis-associated diseases treatment. Furthermore, evidence revealed that athletes’ metabolic biomarkers are improved and demonstrate low chronic inflammatory markers, decreasing morbidity. These findings indicated that an age-appropriate diet along with exercise could be beneficial for decreasing inflammation and age-associated disorders (133–135). Interestingly, the levels of *Akkermansia muciniphila* in the microflora of athletes as well as people with low BMI are higher than people with high BMI (131). *Akkermansia muciniphila* is involved in the destruction of mucosal mucin, possibly improving the function of the intestinal physical barrier. Furthermore, the increase of this bacterial species in the gut microbiome is inversely associated with metabolic disorders, high BMI, and obesity (136, 137).

#### 5.2.4 Obesity and Diabetes

As discussed above, obesity can also be associated with changes in the diversity and composition of the intestinal microbiota. To prove this, a study of two phyla of the gut microbiota in genetically obese mice (ob/ob mice) showed that *Firmicutes* levels increased while *Bacteroidetes* levels decreased (138, 139). Another animal study reported that a high-fat diet was also associated with alterations in intestinal microbiota in mice, which were associated with decreasing the levels of *Eubacterium rectale* and *Blautia coccoides, Bifidobacteria* and Bacteroides-like mouse intestinal bacteria (140, 141).

Moreover, studies showed that the pattern of gut microbiota and dietary-derived microbial metabolites could be associated with type 2 diabetes (T2D) through impact on insulin secretion and sensitivity (142). Among these microbial metabolites, butyrate is produced by the fermentation of dietary fibers. Independent cohort studies on populations with different ethnicities reported that reduced butyrate production by special bacteria spices in gut microbiota had been dependably detected in prediabetes and T2D (143–146). Additionally, current investigations have revealed that following a meal, glucose response is affected by a combination of host physiology and gut microbiota pattern (147, 148). Consequently, gut microbiota analysis and characterization can pave the way for a deeper understanding of the unknown dimensions of diabetes pathogenesis and the emergence of therapeutic approaches by altering the microbiome or eating habits.

### 5.3 Role of Genetics in the Human Gut Microbiota Shaping

To the best of our knowledge, the composition of the human gut microbiome is shaped by several factors, but the relative contribution of the host genetics has not yet been fully elucidated (149, 150). Although some studies reported that the richness of health-related gut bacteria could be influenced by host genetics, analysis of genotype and microbiome data obtained from healthy individuals with several distinct ancestral origins and relatively common environments has shown no significant association between genetic ancestry and gut microbiome pattern. Based on these findings, host genetics have very little effect in determining the composition of the gut microbiome (151). However, in genetically unrelated subjects who share a household, there are considerable similarities in the composition of the microbiomes. Studies have also shown that more than 20% of the inter-person microbiome diversity is associated with anthropometric indices, diet-related factors, and medications.

On the other hand, a comparison of models that use only host environmental and genetic data with data from microbiome analysis has shown that microbiome data increase the predictive accuracy of many human traits, such as criteria related to diabetes and obesity. These clues also suggest that microbiome manipulation to improve clinical consequences may be feasible and effective in various genetic contexts (151). However, the composition of microbiome is principally formed by environmental and non-genetic factors.

### 5.4 Gut Microbiome Products and Colorectal Cancer

Based on the findings of emerging studies using animal models, bacterial metabolites produced by intestinal microbiota can induce tumor progression in CRC by inducing and stimulating the release of genotoxic virulence factors (152–155). Low bacterial diversity and the presence of specific bacterial species in the fecal and intestinal mucus samples obtained from CRC patients have been observed that these bacterial groups can strongly affect mucosal immune responses compared to healthy individuals (156, 157). CRC patients in the early stages of the disease or advanced adenoma have been shown to have different microbiota patterns compared to patients with advanced-stage tumors, suggesting that intestinal microbiota may be involved in tumor progression (158, 159). Significant growth of bacterial species including *F. nucleatum*, *Porphyromonas*, *Bacteroides fragilis*, *Escherichia coli*, *Peptostreptococus*, *Campylobacter*, *Streptococcus gallolyticus*, *Shigella*, and *Enterococus faecalis*, along with a significant reduction in *Rosariabacterium*, *Clostridia*, *Clostridium*, and *Clifridia*, *Roseburia* can reduce butyrate-producing bacteria along with enriching pro-inflammatory opportunistic pathogens that lead to dysbiosis, increase the expression of pro-inflammatory cytokines and increase the risk of tumor cell formation (104, 159–162) (**Figure 1**). According to studies, the abundance of *F. nucleatum* in the intestine is associated with the development of dMMR CRC through an increase in M2 macrophages and a decrease in FOXP3^+^ T cells in the TME (163). *F. nucleatum* is probably involved in tumorigenesis due to the presence of bacterial proteins FadA and Fap2 because bacterial FadA causes tumor formation by activating the WNT/catenin beta 1 signaling pathway, and Fap2 can inhibit NK cells and T cell signaling through binding to the immunoreceptor tyrosine-based inhibition motifs (ITIMs) (163–165).

One of the most important products produced by the gut microbiome is butyrate, which can induce antitumor immune responses and participate in microbiome homeostasis (166). According to the Warburg effect, butyrate decreases the expression of cell cycle-regulating genes and increases the expression of apoptotic genes such as the Fas, which eventually leads to apoptosis (167). Studies in dMMR/MSH mice have shown that butyrate has an inhibitory effect on tumor cell growth. In CT30 cells of mouse colon cancer or SW480 cells of human colon cancer, butyrate also keeps microbial homeostasis by increasing TLR4 expression as well as phosphorylation of the nuclear factor kappa B (NFKB) and mitogen-activated protein kinase (MAPK) (168) (**Figure 1**). Studies on genetically predisposed CRC animal models have shown that microbiota can induce protumorigenic responses by initiating inflammatory signaling pathways (169). Disruption of the balance of anti-inflammatory and inflammatory responses also leads to increased intestinal inflammation, colitis, and eventually adenocarcinoma (170). For example, in IL10 deficient mice, Th1-specific responses to microbiota increased, contributing to intestinal inflammation and tumor formation (170). Another reason for the involvement of microbiota in the growth of tumor cells in CRC is that in germ-free mice, fecal microbiota transplantation from CRC patients leads to the growth of intestinal epithelial cells in the recipient animals (50, 171, 172).

It has been reported that hemostatic disorders occur following the activation of intracellular signaling pathways induced by unfolded proteins that cannot reduce endoplasmic reticulum (ER) stress. Active transcription factor 6 (ATF6) leads to increased ER capacity and degradation of imperfect proteins. Activated ATF6 can also trigger destructive immune responses to gut dysbiosis and increase CRC susceptibility (173, 174).

On the other hand, studies have reported that lysogenic bacteriophages belonging to *Myoviridae* and *Siphoviridae* species can change the bacterial patterns through bacterial lysis, which leads to the production of biofilms by tumor-associated opportunistic species that are anchored to the intestinal epithelium. Following these events, oncogenic bacteria penetrate the intestinal lumen and stimulate cellular transformation and tumor development by inducing inflammatory responses (103, 175).

In CRC, examination of the fungal microbiota metagenome has also shown that in colorectal adenoma biopsies, the fungal genera *Phoma* and *Candida* are more loaded, and these host intestinal fungi pattern alterations may be associated with an increased risk of CRC (176). Moreover, fungal dysbiosis in colon polyps and CRC is associated with an increase in opportunistic fungi *Trichosporon*, *Malassezia*, and *Ascomycota/Basidiomycota* ratio, which may lead to less diversification and dramatic change in microbiota and ultimately induce tumor cell progression in early phases of CRC (176, 177).

Contrary to the mentioned species, some fungi have anti-inflammatory properties and regulate the immune system. In this regard, it has been revealed that *Saccharomyces cerevisiae* could suppress the growth of tumor cells of CRC (HT-29) *via* inducing apoptosis and inhibiting metastasis. Therefore, using *Saccharomyces cerevisiae* might be a potential biological therapeutic strategy in treating CRC (178).

Collectively, all of these studies suggest that in addition to an imbalance in the pattern of intestinal bacteria, changes in gut virome and mycobiome homeostasis can also lead to CRC (**Figure 1** and **Table 1**).

**Table 1:** Effects of different microorganisms on the immune system, the gut microbiome, and immunotherapy.

Microorganism	Effect on the immune system and the gut microbiome	Role in CRC	Effect on immunotherapy of CRC or other malignancies	Ref
***F. nucleatum***	Increase in M2 macrophages and a decrease in FOXP3^+^ T cells in the TME FadA causes tumor formation by activating the WNT/catenin beta 1 signaling pathway Fap2 can inhibit NK cells and T cell signaling through binding to the ITIMs	Pro-tumor		(101–103)
***Myoviridae* and *Siphoviridae***	Bacterial lysis Production of biofilms by tumor-associated opportunistic species Penetrate the intestinal lumen Stimulate cellular transformation Inducing inflammatory responses Tumor development	Pro-tumor		(83, 113)
***Phoma* and *Candida***	Increase in opportunistic fungi (*Trichosporon* and *Malassezia)* Increase *Ascomycota*/*Basidiomycota* ratio Decrease of microbiota diversification	Pro-tumor		(114)
***Saccharomyces cerevisiae***	Anti-inflammatory effects	Anti-tumor		(115)
***Serratia* & *Streptococcus pyogenes***	Anti-inflammatory effects	Anti-tumor		(116, 122, 123)
***Akkermansia muciniphila***	Increase the production of IL12 Recruitment and infiltration of CXCR3^+^, CCR9^+^, CD4^+^ T cells into the mouse tumor site	Anti-tumor	Restoration of the anti- PDCD1 efficacy	(128)
***Bifidobacteria***	Increase DCs function Enhancing antitumor CD8^+^ T cell priming and infiltration in the TME	Antitumor	Combination therapy with CD274 inhibitors and *Bifidobacteria* can nearly completely inhibit the growth and development of melanoma cells	(131)
***Bacteroides* or *B. fragilis***	Improve antitumor immune responses and antitumor CD8^+^ T cells	Antitumor	Improvement of anti-CTLA4 therapy	(19)
***Bacteroides dorei* and *Bacteroides vulgatus***	Nonspecific immune activation	Pro-tumor	Induction of irAEs following immune checkpoint inhibitor therapy in melanoma patients	(133)
***Salmonella typhimurium***	Inhibition of IDO1 Suppression of immunosuppressive responses	Antitumor	shIDO-ST can remarkably reduce the expression and function of IDO Inhibit tumor growth and development in CRC	(137)
***Lactobacillus acidophilus***	Improve antitumor immune responses and antitumor CD8^+^ T cells Improving the gut microbiome homeostasis	Antitumor	Improvement of anti-CTLA4 therapy	(138)
***Ruminococcus* spp. *Alistipes shahiiplayed, Eubacterium limosum*, *Ruthenibacterium lactatiformans*, *F. ulcerans*, *B. uniformis*, *Bacteroides dorei*, *Parabacteroides johnsonii*, *Phascolarctobacterium succinatutens*, *Paraprevotella xylaniphila*, *Alistipes senegalensis*, and *P. gordonii***	Improve antitumor immune responses and antitumor CD8^+^ T cells	Antitumor	Modulating the TME Improvement of immune checkpoint inhibitors effectiveness	(139) (124, 140)
***Colibactin-producing E. coli* (CoPEC)**	Decrease in tumor-infiltrating CD3^+^ T-cells in patients colonized by CoPEC Decrease in CD3^+^ CD8 T-cells in mice with chronic CoPEC infection Increase colonic inflammation Decrease in antitumor T-cells in the mesenteric lymph nodes of CoPEC-infected mice Decrease the anti-PDCD1 immunotherapy efficacy in MC38 tumor model	Pro-tumor		(179)

## 6 Effects of Gut Microbiota in Colorectal Cancer Immunotherapy

Recent investigations have reported that microecology plays a critical role in the effectiveness of CRC treatment by antitumor agents such as chemotherapy as well as immunosuppressive agents (180, 181) (**Table 1**). The gut microbiota has the ability to regulate the antitumor effect of chemotherapy drugs commonly used in chemotherapy to eliminate CRC cancer cells. It has been shown that 5-fluorouracil (5-FU), a chemotherapeutic drug routinely used to treat CRC and other human malignancies, can increase the effectiveness of tumor cell killing under the effects of metabolites produced by gut microbial (182). Correspondingly, eating foods rich in probiotics or supplements containing probiotics, prebiotics, and symbiotics can reduce the risk of CRC (183, 184).

Nowadays, cancer immunotherapy includes approaches such as immuno-binding site-blocking therapy, adoptive immunotherapy, indoleamine 2, 3-dioxygenase 1 (IDO1) inhibitors, cancer vaccines, and nonspecific immunomodulators (180, 185, 186). The combination of cancer immunotherapy and microorganisms was first proposed in the early 19th century, and experimental outcomes showed that the combination of heat-killed *Serratia* and *Streptococcus pyogenes* could effectively increase the survival of sarcoma patients. This increase in survival was probably due to the development of a sustained antitumor immune response (180, 187, 188).

Immune checkpoint inhibitors with PDCD1/CD274 axis inhibitory capability can induce stable clinical outcomes in several patients with malignancy. It has been shown that primary resistance to immunosuppressive inhibitors can be attributed to the abnormal pattern of the gut microbiome because antibiotics can reduce or even inhibit the clinical benefits of immune checkpoint inhibitors in patients with advanced cancer (189). Evidence suggests that immunotherapy is effective in only one subset of people with CRC. In this context, anti-PDCD1 is approved only for use in dMMR/MSH metastatic CRC, which may be due to the expression of higher amounts of neoantigens by this type of CRC. How to respond to the anti-PDCD1 therapy and understand the mechanisms leading to improved immunotherapy performance in dMMR/MSH CRC have not been fully elucidated (190–192).

Moreover, it has been shown that transplantation of fecal microbiota (FMT) from responder cancer patients to immune checkpoint inhibitors to sterile or antibiotic-treated mice potentiates the antitumor effects of PDCD. In contrast, FMT from non-responder patients has no effect on treatment with PDCD1 blockers. Metagenomics studies of at diagnosis patient fecal specimens have disclosed an association between clinical responses to immune checkpoint inhibitors and the relative abundance of *Akkermansia muciniphila*. On the other hand, oral supplementation of non-responder patients with *A. muciniphila* upon FMT restored the anti-PDCD1 efficacy *via* IL12 by increasing the recruitment and infiltration of CXCR3^+^, CCR9^+^, CD4^+^ T cells into the mouse tumor site (193). According to recent studies, the composition of the intestinal flora can predict the effectiveness of allogeneic stem cell transplantation, and this feature confirms that the gut microbiome is effectively involved in the formation of systemic immune responses (194).

As discussed before, gut microbiota can modulate immune responses in the TME. In this context, an investigation on a patient with cutaneous melanoma reported an association between infiltrating CD8^+^ T cells, intratumor bacteria (*Lachnoclostridium*, *Gelidibacter, Flammeovirga*, *Acinetobacter*), and patients’ survival. The outcomes revealed that intratumor *Lachnoclostridium* could modulate CCL5, CXCL9, and CXCL10 levels, affecting the infiltration of CD8^+^ T cells (195). A phase 1 clinical trial was recently performed to evaluate the feasibility and safety of FMT and reinduction of anti-PDCD1 immunotherapy in patients with anti-PDCD1 refractory metastatic melanoma. The findings showed clinical responses in some patients.

Interestingly, patients under FMT treatment experienced promising alterations in expressing several genes involved in antitumor responses and infiltration of effector immune cells in the TME and gut lamina propria (196). Similarly, another clinical trial in this field reported that FMT in combination with anti-PDCD1 in patients with PDCD1 refractory melanoma was well tolerated and accompanied by clinical benefits in about half of these patients. The outcomes demonstrated that responder patients exhibited elevated taxa associated with response to anti-PDCD1 immunotherapy, activation of more effector CD8^+^ T cell, and reduced the number of myeloid cells responsible for release IL8 (197).

In general, the findings of these studies suggest that microbiota may play a key role in regulating the host’s immune system, which is directly related to the success or failure of cancer immunotherapy. As discussed, one of the most important immunotherapy-based approaches in CRC is checkpoint blockers using anti-inhibitory molecules mAbs against CTLA4, PDCD1, and CD274 (198). In this regard, researchers studied mice and showed that *Bifidobacteria* have antitumor properties.

Interestingly, administration of oral *Bifidobacteria* alone can have the same effect as CD274 inhibitors. In addition, combination therapy with CD274 inhibitors and *Bifidobacteria* can nearly completely inhibit the growth and development of cancer cells (199). This treatment can augment DC function, enhancing antitumor T CD8^+^cell priming and infiltration in the TME. As a result, *Bifidobacterium* enhances antitumor immune responses and improves the effectiveness of anti-CD274 immunotherapy through increased trafficking, penetration, and infiltration of effector T CD8^+^ cells into tumor tissue which is considered as one of the most important challenges in cancer immunotherapy.

*Bacteroides* are considered another bacterial species effective in immunotherapy (200). The antitumor property of CTLA4 inhibitors has been documented to be dependent on the presence of *Bacteroides* or *B. fragilis*, and this association may be reciprocal because the administration of CTLA4 inhibitors contributes to the growth of *B. fragilis*. To prove this, it has been shown that sterile mice or mice treated with antibiotics that lacked the itemized bacterial species did not respond to treatment with anti-CTLA4 but responded to this type of treatment by feeding the animals with *B. fragilis* (19). Of course, most of the findings in this field are in malignancies such as melanoma, and perhaps due to the lower response of CRC to checkpoint blockers, the pattern of gut microbiota cannot significantly affect this type of immunotherapy and support it.

In contrast, a recent study on melanoma immunotherapy using immune checkpoint blockers has disclosed that not all patients respond to this treatment, and numerous patients with melanoma who have been treated have potentially life-threatening side effects (irAEs) and some gut microbiota bacterial species such as *Bacteroides dorei* and *Bacteroides vulgatus* may be involved in causing these complications (201).

Recently, an investigation proved that some human malignancies, such as CRC with impaired MMR, but not patients with complete chromosomal mismatch repair, respond to anti-PDCD1 therapy better, probably through the metabolic pathway of glycerol phospholipids (202). These differences in response to anti-PDCD1 therapy could lead to the emergence of therapeutic approaches using gut microbiota, thereby increasing the effectiveness of cancer immunotherapy in CRC patients with MMR by regulating the composition of the intestinal flora (202, 203).

As an anti-inflammatory mediator, IDO1 is also essential for the resistance of host-microbiome homeostasis and is produced by tumor cells and following the activation of TLR4 and TLR9 in DCs that induces immune responses against tumors (204). In the microbiome, IDO1, along with butyrate and short-chain fatty acids (SCFAs), interferes with tryptophan metabolism (189). IDO1 inhibitors may be useful in treating CRC, especially in dMMR/MSH (190, 204). *In vitro* and *in vivo* studies on CRC models of CT26 and MC38 mice have shown the use of attenuated *Salmonella typhimurium* carrying a small hairpin RNA plasmid targeting IDO (shIDO-ST) can remarkably reduce the expression and function of IDO proteins and thereby inhibit tumor growth and development. Moreover, epacadostat was used as a known inhibitor of IDO1, which could also reduce tumor growth by inhibiting IDO1 (205).

In mice models of colon cancer, the use of *Lactobacillus acidophilus* lysate in combination with anti-CTLA4 enhanced antitumor immune responses *via* activation of the TME-infiltrated effector T cells as well as improving homeostasis in the investigated animals (206). These findings suggest that some bacterial species may be very effective in combination therapies with other antitumor agents.

Additionally, it has been disclosed that in mice models of CRC under treatment with immune checkpoint inhibitors, specific bacterial species are associated with promising antitumor responses. In this regard, MC38 cell line treated with anti-PDCD1 or anti-CTLA4 and anti-IL10 plus CpG oligonucleotides demonstrated *Ruminococcus* spp. and *Alistipes shahiiplayed* could induce antitumor responses by modulating the TME (207). Furthermore, other spices including, *Eubacterium limosum*, *Ruthenibacterium lactatiformans*, *F. ulcerans*, *B. uniformis*, *Bacteroides dorei*, *Parabacteroides johnsonii*, *Phascolarctobacterium succinatutens*, *Paraprevotella xylaniphila*, *Alistipes senegalensis*, and *P. gordonii* might be beneficial in the improvement of immune checkpoint inhibitors’ effectiveness (189, 208).

A study on the effect of antibiotic therapy on adoptive cell therapy showed that antibiotics inhibited the innate immune system responses in irradiated mice and reduced the effectiveness of adoptive cell therapy. In CRC mouse models, the use of broad-spectrum antibiotics reduced the effectiveness of CD4^+^ T cell transplantation against implanted colorectal tumor cells (209). Only one animal study on the effect of antibiotics on CAR-T cell therapy reported that broad-spectrum antibiotics had not reduced the survival of BA20-lymphoma mice treated with CD19 CAR-T cells (209). However, the change and decrease in gut microbiome pointedly improved the duration of B cells aplasia as well as the persistence of CAR-T cells in these mice (210).

## 7 Concluding Remarks

According to studies on the role of gut microbiota in tumorigenesis and also the antitumor activity of some bacterial species, it can be concluded that the gut microbiome can be of great importance in CRC immunopathogenesis by regulating immune responses. On the other hand, CRC immunotherapy has been developed in various ways, and in the case of CRC immune-checkpoint blockers, they have been studied more than other immunotherapy approaches and have had relatively promising effectiveness. However, this type of immunotherapy also faces many challenges. Therefore, the use of some bacterial species to change the pattern of the intestinal microbiome may increase the effectiveness of immunotherapy with checkpoint blockers, which can be very useful in treating CRC patients. Nevertheless, due to the complexity of microbiomes and the high diversity of microorganisms, including bacteria, viruses and fungi, and their unknown functions involved in tumorigenesis or antitumor mechanisms, further studies to identify the function of each of them and also use these microorganisms in combination with other therapeutic approaches appear to be essential.

## Author Contributions

YL: Conception, design and inviting co-authors to participate. Y-NZ: Writing original manuscript draft. D-XK: Review and editing of manuscript critically for important intellectual content and provided comments and feedback for the scientific contents of the manuscript. All authors read, revised and approved the final manuscript.

## Conflict of Interest

The authors declare that the research was conducted in the absence of any commercial or financial relationships that could be construed as a potential conflict of interest.

## Publisher’s Note

All claims expressed in this article are solely those of the authors and do not necessarily represent those of their affiliated organizations, or those of the publisher, the editors and the reviewers. Any product that may be evaluated in this article, or claim that may be made by its manufacturer, is not guaranteed or endorsed by the publisher.
